# Knowledge Distillation and Explainability Analysis for Lightweight Retinal Disease Classification Using MultiEYE Fundus Images

**DOI:** 10.3390/diagnostics16182945

**Published:** 2026-09-11

**Authors:** Gülşen Türker, Arif Koyun

**Affiliations:** Department of Computer Engineering, Faculty of Engineering and Natural Sciences, Suleyman Demirel University, 32260 Isparta, Turkey; arifkoyun@sdu.edu.tr

**Keywords:** retinal disease classification, fundus images, knowledge distillation, EfficientNet-B0, ConvNeXtV2, Grad-CAM, MultiEYE

## Abstract

**Background/Objectives**: Class imbalance and probabilistic prediction quality are important considerations in lightweight retinal classification. We evaluated whether knowledge distillation (KD) from a ConvNeXtV2-Base teacher improved an EfficientNet-B0 student in nine-class MultiEYE fundus classification. **Methods**: Exact-content screening quarantined 131 of 58,036 records because of exact-content duplication or label conflicts. Six prespecified seeds were evaluated in no-KD, label-smoothing, and KD arms (18 runs) under matched training conditions. Within the controlled experiment, TEST data were not used for training, hyperparameter tuning, checkpoint or model selection, or protocol modification. The primary endpoint was the KD−no-KD macro-F1 difference on decontaminated TEST (*n* = 11,573), assessed by paired image-level bootstrap (10,000 replicates). Grad-CAM and Grad-CAM++ were examined qualitatively using one deterministically selected case per class and six-seed consensus maps. **Results**: Mean macro-F1 was 0.593, 0.597, and 0.633 for no-KD, label smoothing, and KD. KD exceeded no-KD by 0.039 (95% CI, 0.025–0.053; *p* < 0.001), with positive paired differences in all six seeds, and label smoothing by 0.036 (Holm-adjusted *p* < 0.001); label smoothing did not differ from no-KD (Holm-adjusted *p* = 0.554). Class-wise F1 differences were non-negative across all nine classes, with six remaining significant after correction for multiple comparisons. The Brier score difference was −0.077 (95% CI, −0.081 to −0.072; Holm-adjusted *p* < 0.001), while ECE decreased descriptively from 0.171 to 0.084. Nine-class Grad-CAM and direct Grad-CAM++ consensus maps enabled qualitative no-KD versus KD comparison without establishing lesion-localization superiority. **Conclusions**: KD improved macro-F1 and lowered the Brier score while retaining the same EfficientNet-B0 student at inference. The evaluated label-smoothing configuration did not reproduce the macro-F1 gain; clinical superiority and external generalizability remain unestablished.

## 1. Introduction

Diabetic retinopathy (DR), glaucoma, and age-related macular degeneration (AMD) are important ocular diseases associated with visual impairment and blindness worldwide [1]. Color fundus photography enables visualization of key retinal structures, including the optic disc, macula, and retinal vasculature, as well as visible abnormalities associated with retinal disease [2]. Deep learning has enabled automated analysis of fundus photographs for DR and related ocular diseases, including possible glaucoma and AMD [3]. Prospective evaluation of an autonomous artificial intelligence system for DR detection in primary-care settings has also demonstrated the feasibility of image-based diagnostic assessment outside specialist ophthalmology services [4].

Retinal disease classification can be complicated by marked class imbalance. Transformer-based [5] and CNN-based approaches incorporating squeeze-and-excitation modules [6] have been investigated for multi-label retinal disease classification, and long-tailed retinal classification under class-imbalanced distributions has also been specifically addressed [7]. Although the present study considers a nine-class single-label task, the MultiEYE fundus data are likewise markedly imbalanced across disease categories. Accordingly, macro-F1 was specified as the primary performance endpoint, complemented by overall and class-wise performance measures.

Model compactness is another consideration in automated retinal classification. Previous studies have investigated lightweight deep learning models [8], lightweight CNNs with attention mechanisms [9], intermediate-feature reduction in compact fundus classifiers [10], and CNN–vision-transformer hybrid architectures designed to balance predictive performance and computational requirements [11]. Knowledge distillation (KD) provides a different strategy in which a teacher model guides student training, commonly using temperature-scaled teacher probabilities as soft targets alongside ground-truth labels [12]. KD has also been investigated as a model-compression approach in medical machine learning [13]. In the present controlled comparison, ConvNeXtV2-Base [14] and EfficientNet-B0 [15] were used as the teacher and student architectures, respectively. The EfficientNet-B0 architecture was identical across the no-KD, label-smoothing, and KD conditions, allowing the effect of the training strategy to be evaluated without changing the student’s inference-time architecture or parameter count.

The MultiEYE dataset provides a relevant setting for this comparison because it includes a nine-class fundus component together with OCT data in a common disease label space [16]. In the original MultiEYE study, OCT-CoDA used an OCT-based teacher to guide a fundus student during training [16]. Related retinal KD studies have examined fundus-to-OCT knowledge transfer [17] and cross-modal distillation combined with quantization-aware training [18]. In contrast, the present study addresses a within-modality setting in which OCT images were not used for teacher or student training, checkpoint selection, or inference. Knowledge transfer was restricted to a fundus-to-fundus teacher–student framework, while the student architecture and imaging modality were held constant across the principal experimental conditions.

Data integrity was also considered because screening of the distributed MultiEYE fundus data identified byte-identical image content across partition boundaries and identical content associated with conflicting labels. Deterministic exact-content-decontaminated derivative TRAIN, DEV, and TEST views were therefore used for the controlled experiments while preserving the original split assignments; this procedure does not establish patient-level or eye-level independence or exclude perceptually similar near-duplicates. Grad-CAM [19] and Grad-CAM++ [20] were used only for qualitative comparison of model-activation patterns. Because no lesion-level reference annotations were used for localization validation, the resulting maps were not interpreted as evidence of anatomical correctness or clinically validated lesion localization.

The primary objective of the present study was to determine whether output-level KD from a ConvNeXtV2-Base teacher improved macro-F1 for a fixed EfficientNet-B0 student in nine-class MultiEYE fundus classification. A label-smoothing condition was included as an active regularization comparator to determine whether the KD-associated effect was reproduced by fixed target smoothing alone. Probabilistic performance was evaluated as a secondary component of model assessment. The primary hypothesis was that teacher-guided KD would improve macro-F1 relative to the same EfficientNet-B0 architecture trained without distillation while leaving the student inference architecture and parameter count unchanged.

## 2. Materials and Methods

**Figure 1:**
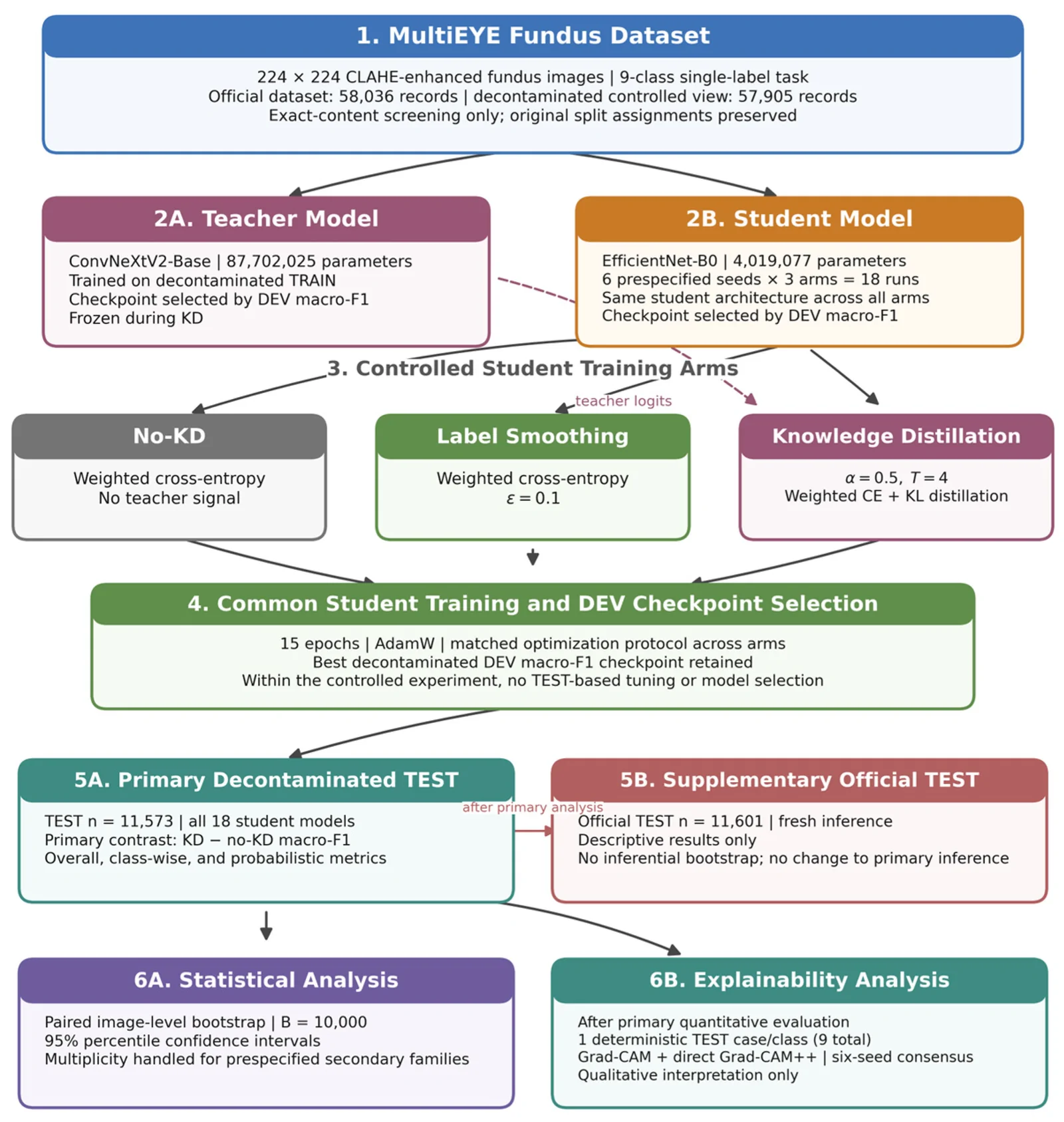
Proposed knowledge distillation-based study framework.

The overall experimental workflow of this study is summarized in Figure 1. Starting from the official MultiEYE fundus split, exact-content-decontaminated TRAIN, DEV, and TEST views were constructed. A pretrained ConvNeXtV2-Base teacher was fine-tuned on the decontaminated TRAIN view and selected using the corresponding DEV view. The fixed EfficientNet-B0 student architecture was subsequently trained under no-KD, label-smoothing, and KD conditions, with the teacher held fixed during distillation.

### 2.1. Dataset, Preprocessing, and Data Integrity

The experiments were conducted using the nine-class fundus component of the MultiEYE dataset [16]. The classification task comprised Normal, dry age-related macular degeneration (dAMD), central serous chorioretinopathy (CSC), diabetic retinopathy (DR), glaucoma (GLC), epiretinal membrane (ERM; corresponding to the “Macular Epiretinal Membrane” [MEM] class in the original MultiEYE dataset), myopia (MYO), retinal vein occlusion (RVO), and wet age-related macular degeneration (wAMD). The dataset code MEM is retained in tables for consistency with the source data. The official fundus split contained 58,036 records: 34,834 in TRAIN, 11,601 in DEV, and 11,601 in TEST.

Before model training in the controlled study, data integrity was assessed at the exact-content level using SHA-256 digests computed from the full image files. The audit identified 97 non-singleton SHA-256 groups comprising 201 records. Twenty groups involving 43 records crossed official partition boundaries. Eleven groups contained conflicting class labels, including four cross-partition groups. Among the 77 duplicate groups confined to individual partitions, seven contained conflicting labels, whereas 70 were same-label groups comprising 142 records. A systematic duplication in DEV involved 20 GLC records duplicated once, resulting in 40 records; the 20 redundant copies were quarantined. Four additional DEV GLC records belonged to cross-partition conflicting-label groups. Consequently, DEV GLC support decreased from 134 to 110 after the application of the decontamination policy.

A deterministic, model-result-blind decontamination policy was applied before the controlled model experiments. Any group of records sharing an exact SHA-256 digest across more than one official partition was quarantined in its entirety, with cross-partition quarantine taking precedence over within-partition rules. For same-label duplicate groups confined to a single partition, the record with the smallest original source-list line number was retained, and the remaining copies were quarantined. Any within-partition group in which identical image content was associated with conflicting class labels was quarantined in its entirety. Records were not reassigned among TRAIN, DEV, and TEST. The official split files were preserved unchanged, and derivative exact-content-decontaminated views were generated for controlled teacher and student training, decontaminated-DEV checkpoint selection, and primary exact-content-decontaminated TEST evaluation.

Application of these rules quarantined 131 of the 58,036 records and retained 57,905 records: 34,792 in TRAIN, 11,540 in DEV, and 11,573 in TEST. Specifically, 43 records belonging to cross-partition groups, 16 records belonging to within-partition conflicting-label groups, and 72 redundant copies from within-partition same-label duplicate groups were quarantined. The resulting class distributions are shown in Table 1.

Class proportions differed across the official partitions, and these differences remained after exact-content decontamination because records were not reassigned among TRAIN, DEV, and TEST.

The controlled experiments used the 224 × 224 CLAHE-enhanced color fundus images provided in the distributed data package. MultiEYE reports contrast-limited adaptive histogram equalization (CLAHE) as part of its fundus-image preprocessing [16]; CLAHE was not reapplied in the present study. For controlled student training, images were loaded in RGB format and augmented using random horizontal flipping (*p* = 0.5), random rotation within ±10°, and color jitter with brightness = 0.10, contrast = 0.10, saturation = 0.05, and hue = 0. Images were then normalized using the ImageNet channel means [0.485, 0.456, 0.406] and standard deviations [0.229, 0.224, 0.225]. DEV and TEST preprocessing used the same resize and normalization steps without stochastic augmentation.

Marked class imbalance remained after exact-content decontamination: Normal and DR accounted for most observations, whereas CSC and MEM were sparsely represented. Accordingly, macro-F1 was prespecified as the primary performance endpoint, supplemented by overall and class-wise metrics. Class weights for controlled student training were calculated exclusively from the decontaminated TRAIN partition; the weighting formulation is described in the student-training section.

Throughout this study, the terms clean and decontaminated refer specifically to the control of byte-identical image content under the SHA-256-based procedure described above. They do not imply patient-level or eye-level independence because complete and reliably verifiable patient or eye identifiers were unavailable for all records. The procedure also did not identify or remove visually similar or near-duplicate images that were not byte-identical. Accordingly, no claim of patient-level, eye-level, or near-duplicate independence is made.

During the controlled experiments, TEST data were not used for teacher or student training, hyperparameter tuning, checkpoint or model selection, or protocol modification. Teacher and student model development was based exclusively on the decontaminated TRAIN and DEV partitions. The TEST partition was used only after model selection for final evaluation.

### 2.2. Model Architectures and Knowledge Distillation

ConvNeXtV2-Base [14] was used as the higher-capacity teacher architecture, and EfficientNet-B0 [15] as the student architecture. EfficientNet coordinates changes in network depth, width, and input resolution within a single scaling framework [15]. ConvNeXt V2 retains a convolution-only design and incorporates architectural changes, including Global Response Normalization (GRN), together with an FCMAE-based pre-training framework [14]. Both architectures were fixed before the controlled student experiments. The EfficientNet-B0 student was initialized using pretrained weights, with its classifier adapted to the nine-class task; the resulting model contained 4,019,077 parameters. The teacher used the pretrained convnextv2_base.fcmae_ft_in22k_in1k configuration and contained 87,702,025 parameters. It was fine-tuned on the exact-content-decontaminated TRAIN view, with the checkpoint selected by the highest macro-F1 on the corresponding DEV view. The selected teacher was then frozen and used unchanged across all six KD runs. A single teacher-specific training configuration was used, with class-weighting and training hyperparameters distinct from those used for student training.

The same EfficientNet-B0 architecture was evaluated under three controlled training conditions: no knowledge distillation (no-KD), label smoothing, and knowledge distillation (KD). For each prespecified seed, all three conditions started from the same seed-specific initial model state. Data identity across conditions was also verified. Within each seed, the full sequence of training image references was identical across the three conditions in all 15 epochs. In addition, the augmented-input tensor and label hashes from the first three batches of each epoch matched across conditions. These verification criteria were satisfied for all six prespecified seeds, and no performance metric was used in the pairing verification. The label-smoothing condition served as a teacher-independent regularization comparator, motivated by prior work examining the relationship between label smoothing and knowledge distillation [21].

In the no-KD condition, the student was optimized using class-weighted cross-entropy. The label-smoothing condition used the same class weights with a smoothing coefficient of *ε* = 0.1 and did not use teacher outputs. In the KD condition, the same class-weighted cross-entropy formulation served as the supervised component and was combined with a teacher-derived KL-divergence term. In the full KD objective, the supervised and distillation components were weighted by *α* and 1 − *α*, respectively. Class weights were applied only to the supervised cross-entropy component and not to the KL-divergence term. The teacher had been trained using direct inverse-frequency class weighting, whereas the student supervised loss used a separate square-root-based class-weighting scheme derived from the clean TRAIN partition. Because the teacher outputs entered the distillation term, the present design does not isolate the specific contribution of the teacher’s class-weighting scheme from other information encoded in those outputs.

For input sample *i*, let *z_t,i_* and *z_s,i_* denote the teacher and student logit vectors, respectively. Following the conventional temperature-based formulation of KD [12], the corresponding class-probability distributions were defined as

(1) $$p_{t,i}^{\left(T\right)}=softmax\left(\frac{z_{t,i}}{T}\right),$$

and

(2) $$p_{s,i}^{\left(T\right)}=softmax\left(\frac{z_{s,i}}{T}\right).$$

Here, *T* denotes the temperature parameter. The distillation loss used in this study was calculated as

(3) $$L_{distill}=\frac{T^{2}}{B}\sum_{i=1}^{B}D_{KL\;}\left(p_{t,i}^{\left(T\right)}∥p_{s,i}^{\left(T\right)}\right),$$

where *B* denotes the mini-batch size. The *T*^2^ factor compensates for the temperature-dependent scaling of the gradients associated with the soft-target term in conventional KD [12]. The total objective optimized in the KD arm was defined as

(4) $$L_{KD}=\alpha L_{WCE}+\left(1-\alpha \right)L_{distill},$$

where *L*_WCE_ denotes class-weighted cross-entropy computed from the reference labels, and *D*_KL_ denotes the Kullback–Leibler divergence. The temperature and mixing coefficient were fixed at *T* = 4 and *α* = 0.5, respectively, for all KD runs. These values were not re-optimized during the controlled analysis; their development-stage selection is reported separately in Section 3.1. No class weighting was applied to the KL-divergence term. Accordingly, the KD-versus-no-KD comparison evaluates the effect of replacing the no-KD training objective with the complete prespecified KD objective, which combines a rescaled supervised term with teacher-derived distillation. It should therefore not be interpreted as estimating the isolated contribution of the KL-divergence term.

The teacher was required only during KD training and was not used for student inference. Consequently, knowledge distillation did not alter the EfficientNet-B0 inference architecture or increase the student’s parameter count.

### 2.3. Training Protocol

The controlled student experiment comprised 18 runs across three training arms (no-KD, label smoothing, and KD) and six prespecified random seeds (42, 3407, 7311, 12011, 19023, and 26081). The seed set, arm definitions, training settings, checkpoint-selection rule, and run order were fixed before the 18 full training runs. Each of the six possible arm orders was used once across the six seeds. Within each seed, all three arms started from the same seed-specific hash-locked EfficientNet-B0 initialization. Training-data identity was verified across arms. For every seed and epoch, the full sequence of training image references was identical across the three arms. The augmented-input tensor and label hashes from the first three batches of each epoch also matched across arms. These pairing checks were satisfied for all six seeds, and no performance metric was used in the pairing verification. Student performance was not used to alter the seed set, arm order, training configuration, or fixed training duration.

All EfficientNet-B0 students were trained for 15 fixed epochs with a batch size of 32 using AdamW [22]. The initial learning rate was 3 × 10^−4^, with a weight decay of 1 × 10^−4^, *β*_1_ = 0.9, *β*_2_ = 0.999, and *ε* = 10^−8^; AMSGrad was disabled. The learning rate was updated using cosine annealing over the 15-epoch training period (*T*_max_ = 15, *η*_min_ = 0), following the cosine annealing form described in SGDR [23] but without warm restarts. Mixed-precision training with CUDA 12.8 float16 and loss scaling was used [24]; the implementation employed PyTorch (version 2.11.0+cu128) automatic mixed precision with GradScaler. Early stopping and gradient clipping were not applied. Four data-loading workers were used with persistent workers enabled, and incomplete final minibatches were retained (drop_last = False). The fixed training duration gave all three arms the same number of training epochs and checkpoint-selection opportunities. For each run, the checkpoint with the highest macro-F1 on the decontaminated DEV view was retained; an exact tie was resolved in favor of the earlier epoch.

Student class weights were calculated exclusively from the decontaminated TRAIN partition and were identical across the three arms. For class *c*,

(5) $$w_{c}=clip\left(\frac{\sqrt{\frac{N}{Kn_{c}}}}{\frac{1}{K}\sum_{j=1}^{K}\sqrt{\frac{N}{Kn_{j}}}},0.25,5.00\right)$$

Here, *N* = 34,792 is the number of decontaminated TRAIN records, *K* = 9 is the number of classes, and *n_c_* denotes the TRAIN support for class *c*. The square-root-transformed weights were normalized by their mean before clipping, and no renormalization was performed after clipping. The resulting weights were 0.250, 0.619, 2.195, 0.250, 0.842, 1.664, 1.164, 1.099, and 1.053 for Normal, dAMD, CSC, DR, GLC, MEM, MYO, RVO, and wAMD, respectively. The implementation computes the weights by taking the square root of *N*/(*Kn_c_*), dividing by the mean across classes, and then clipping to [0.25, 5.0], with no post-clipping renormalization.

The ConvNeXtV2-Base teacher was fine-tuned separately on the decontaminated TRAIN view using seed 42. Training was performed for 10 fixed epochs with a batch size of 16 using AdamW [22], with a learning rate of 5 × 10^−5^, weight decay of 1 × 10^−4^, *β*_1_ = 0.9, *β*_2_ = 0.999, and *ε* = 10^−8^; AMSGrad was disabled. The learning rate was updated using cosine annealing over the 10-epoch training period (*T*_max_ = 10, *η*_min_ = 0), without warm restarts. Teacher augmentation consisted of random horizontal flipping (*p* = 0.5) and random rotation within ±10°, without color jitter. Class weights were calculated from the decontaminated TRAIN partition using direct inverse-frequency weighting, *w_c_* = *N*/(*Kn_c_*), rather than the square-root-transformed and clipped weighting scheme used for the student models. Mixed-precision training with CUDA 12.8 float16 and loss scaling was used; the implementation employed PyTorch (version 2.11.0+cu128) automatic mixed precision with GradScaler. Data loading used num_workers = 0, and incomplete final minibatches were retained. Neither early stopping nor gradient clipping was applied. The checkpoint with the highest macro-F1 on the decontaminated DEV view was retained, with exact ties resolved in favor of the earlier epoch. The selected teacher checkpoint was then frozen and used unchanged across all six KD runs.

### 2.4. Evaluation and Statistical Analysis

Primary evaluation was performed on the exact-content-decontaminated TEST view (*n* = 11,573). Performance and calibration metrics were calculated separately for each of the six prespecified seeds and summarized as the mean ± sample standard deviation. Macro-F1 was the prespecified primary performance endpoint, with KD versus no-KD as the primary contrast. Additional measures included accuracy, weighted-F1, macro-precision, macro-recall, class-wise precision, recall, F1, and support, macro-AUROC, macro-average precision (macro-AP), multiclass Brier score, and expected calibration error (ECE). Macro-AUROC was calculated as the unweighted mean of the nine one-versus-rest AUROC values, and macro-AP as the unweighted mean of the nine class-wise average-precision values. The multiclass Brier score was calculated as the mean across images of the summed squared differences between the predicted class probabilities and the corresponding one-hot reference labels. ECE was calculated as a top-label measure using 15 approximately equal-mass bins and the maximum softmax probability as the confidence score. ECE was reported descriptively; the multiclass Brier score was included in the clinical/calibration inferential family described below.

Before controlled TEST evaluation, a predefined exploratory DEV-only logit-adjustment sensitivity analysis was performed using the class-prior approach described by Menon et al. [25]. For class *c*, the adjusted logit was defined as $z_{c}^{\prime }=z_{c}-\tau \log\pi_{c}$, where *π_c_* was estimated exclusively from the decontaminated TRAIN partition. Candidate values were *τ* ∈ {0, 0.5, 1.0}. A single *τ* was selected for each arm according to the highest mean DEV macro-F1 across the six seeds, with exact ties resolved in favor of the smaller value. The selected value was *τ* = 0 for all three arms; therefore, the primary TEST evaluation used the original, unadjusted logits. TEST performance was not used in this selection, and no best-seed selection was performed.

Statistical inference used paired image-level bootstrap resampling with (*B* = 10,000) replicates [26]. Within each replicate, a single set of resampled image indices was applied identically to the outputs of all 18 runs, preserving image-wise pairing across the model outputs. Metrics were calculated separately for each seed and arm; arm differences were formed within each seed, and the replicate-level effect was defined as the arithmetic mean of the six seed-specific paired differences. Two-sided 95% percentile confidence intervals were obtained from the 2.5th and 97.5th percentiles of the bootstrap effect distribution. Seeds were not resampled, and no hierarchical bootstrap was performed; accordingly, these intervals quantify image-level sampling uncertainty conditional on the six prespecified trained seeds.

Two-sided centered-bootstrap Monte Carlo *p*-values were calculated from the bootstrap distribution. A + 1 finite-simulation correction was applied to the exceedance count [27]:

(6) $$p=\frac{1+\sum_{b=1}^{B}1(\left|\theta_{b}-\hat{\theta }\right|\geq \left|\hat{\theta }\right|)}{B+1}$$

Here, $\hat{\theta }$ denotes the observed effect and θ_b_ the effect obtained in bootstrap replicate b. With *B* = 10,000, the minimum attainable *p*-value was 1/10,001 ≈ 0.00009999. The primary KD-versus-no-KD macro-F1 comparison was not adjusted for multiplicity.

Multiplicity correction was defined before controlled TEST inference for the secondary inferential families. The label-smoothing-versus-no-KD and KD-versus-label-smoothing macro-F1 contrasts formed a two-comparison Holm-adjusted family [28]. The KD-versus-no-KD differences in DR F1, GLC F1, and multiclass Brier score formed a separate Holm-adjusted family [28]. The nine class-wise KD-versus-no-KD F1 comparisons were adjusted using the Benjamini–Hochberg false-discovery-rate procedure [29], with all nine classes retained in the family. Because CSC and MEM had limited TEST support (*n* = 30 and *n* = 42, respectively), their class-wise results were interpreted together with support counts, effect estimates, and confidence-interval widths rather than on adjusted significance labels alone. All other metrics were treated descriptively and were not subjected to additional post hoc inferential testing.

Within the controlled study, TEST data were not used for training, hyperparameter tuning, checkpoint or model selection, logit-adjustment selection, or protocol modification. The official TEST partition (*n* = 11,601) was evaluated descriptively by fresh inference across all 18 models only after the primary decontaminated-TEST analysis had been completed and locked. For this supplementary evaluation, fresh inference was performed across all 18 models, without bootstrap hypothesis testing, multiplicity correction, or definition of any additional hypothesis family. The official TEST evaluation therefore did not contribute to the primary statistical inference.

### 2.5. Explainability Analysis

After completion of the prespecified quantitative evaluation, an additional post hoc qualitative explainability analysis was conducted across all nine retinal classes. One case per class was selected from the decontaminated TEST view using a deterministic preserved-order middle-floor rule, ⌊(*m*−1)/2⌋, where *m* denotes the class support. This nine-class selection rule was defined after the primary analysis had been locked but before model outputs or heatmaps were examined for this extension. Case selection was independent of model predictions, correctness, confidence scores, and heatmap appearance.

Grad-CAM [19] and direct Grad-CAM++ [20] were applied to all 18 student models from the controlled experiment. The Grad-CAM++ implementation used the location-specific weighting of positive gradients described by Chattopadhyay et al. [20]. Attributions targeted the raw logit of the reference-label class and used the output of the top-level EfficientNet-B0 *bn2* layer as the feature tensor. For each case, training arm, and attribution method, positive native feature-resolution class activation maps from the six prespecified seeds were independently min–max normalized and averaged to obtain a six-seed consensus map. The principal qualitative comparison was between no-KD and KD; label-smoothing maps were generated but were not part of the principal comparison. Absolute heatmap intensities were not compared across panels because normalization was performed independently. The maps were interpreted only as qualitative visualizations of model-activation patterns and not as evidence of anatomical correctness or clinically validated lesion localization.

### 2.6. Use of Generative Artificial Intelligence

ChatGPT (GPT-5.6 Sol; OpenAI) was used for limited assistance with selected programming code drafting and technical wording. All AI-assisted outputs were reviewed and verified by the authors. All model training runs and computational analyses were executed by the authors, and all reported numerical results were derived from the authors’ experimental runs.

## 3. Results

Based on the architecture-selection analyses, ConvNeXtV2-Base and EfficientNet-B0 were selected as the teacher and student architectures, respectively. A new ConvNeXtV2-Base teacher was initialized with pretrained weights and fine-tuned on the decontaminated TRAIN partition; the checkpoint with the highest decontaminated DEV macro-F1 was selected and subsequently held fixed for all KD runs. The controlled experiment compared no-KD, label smoothing, and KD training of EfficientNet-B0 across six fixed random seeds, with final performance evaluated on the decontaminated TEST view.

### 3.1. Development-Set Screening and Model Selection

An initial historical development-stage screening was used to select the teacher and student model families and the KD settings summarized in Table 2. Among the evaluated lightweight student architectures, EfficientNet-B0 achieved the highest DEV macro-F1 (0.6208), compared with MobileNetV3-Large (0.5641) and EfficientNetV2-B0 (0.5286), and was therefore selected as the student architecture.

Among the evaluated teacher architectures, ConvNeXtV2-Base achieved the highest DEV macro-F1 (0.7098), followed by ConvNeXt-Small (0.6880), ConvNeXt-Tiny (0.6690), and RETFound-MAE (0.6176). ConvNeXtV2-Base was therefore selected as the teacher architecture. Weighted cross-entropy achieved the highest DEV macro-F1 in the development-stage loss comparison and was retained for subsequent student training. Among the evaluated KD configurations, the combination of *T* = 4 and *α* = 0.5 achieved the highest DEV macro-F1 (0.6685) and was carried forward without re-optimization. These screening results were obtained on the original DEV split before exact-content decontamination and are reported only to document the historical model- and hyperparameter-selection process; they were not used as performance estimates in the subsequent controlled KD comparison.

For the controlled experiment, a ConvNeXtV2-Base teacher initialized with pretrained weights was fine-tuned on the decontaminated TRAIN partition. Checkpoint selection was based exclusively on decontaminated DEV macro-F1. The highest DEV macro-F1 was achieved at epoch 9 (0.6743), and the corresponding checkpoint was subsequently held fixed as the teacher for all six KD runs.

### 3.2. Overall Classification Performance

On the decontaminated TEST view (*n* = 11,573), KD yielded the highest mean value for each of the overall performance measures reported in Table 3. Accuracy was 0.7534 ± 0.0036 for no-KD, 0.7569 ± 0.0035 for label smoothing, and 0.7830 ± 0.0028 for KD. The corresponding macro-F1 values were 0.5931 ± 0.0181, 0.5965 ± 0.0126, and 0.6325 ± 0.0135, respectively, while weighted-F1 was 0.7403 ± 0.0043, 0.7452 ± 0.0048, and 0.7715 ± 0.0033. KD also showed the highest mean macro-recall (0.6354 ± 0.0144), macro-precision (0.6917 ± 0.0128), macro-AUROC (0.9453 ± 0.0012), and macro-AP (0.6688 ± 0.0096).

In the paired bootstrap analysis, the mean KD-versus-no-KD difference for the primary macro-F1 endpoint was +0.0394 (95% CI, 0.0252–0.0534; *p* < 0.001). Label smoothing differed from no-KD by +0.0035 (95% CI, −0.0081 to 0.0153; Holm-adjusted *p* = 0.554), whereas KD exceeded label smoothing by +0.0360 (95% CI, 0.0239–0.0479; Holm-adjusted *p* < 0.001). The KD-versus-no-KD mean differences in accuracy and weighted-F1 were +0.0296 and +0.0312, respectively; these two differences are reported descriptively because no additional inferential tests were performed for these measures.

### 3.3. Class-Wise Performance

Class-wise KD-versus-no-KD F1 differences varied in magnitude and precision across the nine retinal classes (Table 4). Point estimates were positive for all classes. The largest estimated difference was observed for MEM (+0.1365; 95% CI, 0.0770–0.2015), followed by DR (+0.0485; 95% CI, 0.0429–0.0544), dAMD (+0.0434; 95% CI, 0.0102–0.0770), CSC (+0.0341; 95% CI, −0.0580 to 0.1215), and GLC (+0.0335; 95% CI, 0.0043–0.0624). Smaller positive point estimates were observed for Normal (+0.0214), RVO (+0.0209), wAMD (+0.0154), and MYO (+0.0014).

After Benjamini–Hochberg false-discovery-rate adjustment across all nine class-wise comparisons, the KD-versus-no-KD F1 differences were statistically supported for Normal, dAMD, DR, GLC, MEM, and wAMD. In contrast, the confidence intervals for CSC, MYO, and RVO included zero, with BH-adjusted *p*-values of 0.4996, 0.8727, and 0.0935, respectively. The comparatively large point estimate for MEM should be interpreted in the context of its limited TEST support (*n* = 42). Similarly, the CSC estimate was imprecise, with only 30 TEST records and a wide confidence interval. These findings therefore indicate positive class-wise point estimates with heterogeneous uncertainty rather than uniform evidence of improvement across all retinal classes.

### 3.4. Inferential Comparisons and Calibration

The primary KD-versus-no-KD comparison yielded a mean macro-F1 difference of +0.0394 (95% CI, 0.0252–0.0534; *p* < 0.001; Table 5). Because this contrast represented the primary inferential comparison, no multiplicity adjustment was applied. Two additional macro-F1 contrasts were evaluated as a separate hypothesis group with Holm adjustment. The label-smoothing-versus-no-KD contrast was +0.0035 (95% CI, −0.0081 to 0.0153; Holm-adjusted *p* = 0.554), whereas the KD-versus-label-smoothing contrast was +0.0360 (95% CI, 0.0239–0.0479; Holm-adjusted *p* < 0.001).

A separate prespecified group comprised the KD-versus-no-KD comparisons for DR F1, GLC F1, and multiclass Brier score, with Holm adjustment applied across these three tests. The mean F1 difference was +0.0485 for DR (95% CI, 0.0429–0.0544; Holm-adjusted *p* < 0.001) and +0.0335 for GLC (95% CI, 0.0043–0.0624; Holm-adjusted *p* = 0.0259). The mean multiclass Brier-score difference was −0.0767 (95% CI, −0.0812 to −0.0722; Holm-adjusted *p* < 0.001), indicating a lower Brier score with KD. The DR and GLC contrasts reported here belong to this separately defined three-test group and are distinct from the nine-class Benjamini–Hochberg analysis reported in Section 3.3.

Across the six seeds, mean multiclass Brier scores were 0.4113 ± 0.0100 for no-KD, 0.4089 ± 0.0047 for label smoothing, and 0.3346 ± 0.0033 for KD. The corresponding ECE values were 0.1708 ± 0.0101, 0.1272 ± 0.0078, and 0.0835 ± 0.0069, respectively. ECE was defined as a descriptive measure and was not subjected to inferential testing; therefore, between-arm differences in ECE are reported descriptively and are not interpreted as statistically supported effects.

After completion of the primary decontaminated-TEST analysis, descriptive evaluation on the official MultiEYE TEST partition (*n* = 11,601) yielded a mean accuracy of 0.7832 ± 0.0028 and a mean macro-F1 of 0.6325 ± 0.0135 across the six KD runs.

### 3.5. Explainability Results

After completion of the prespecified quantitative evaluation, a separate post hoc qualitative explainability analysis was conducted across all nine retinal classes. One case per class was selected deterministically from the preserved within-class order of the decontaminated TEST view using the middle-position rule defined for this analysis. Case selection did not use model predictions, prediction correctness, logits, probabilities, confidence scores, quantitative performance results, statistical outcomes, or heatmap appearance. Grad-CAM and direct Grad-CAM++ maps were generated for all 18 student models, and six-seed consensus maps were constructed for each training arm. The principal qualitative comparison was restricted to the no-KD and KD EfficientNet-B0 models.

**Figure 2:**
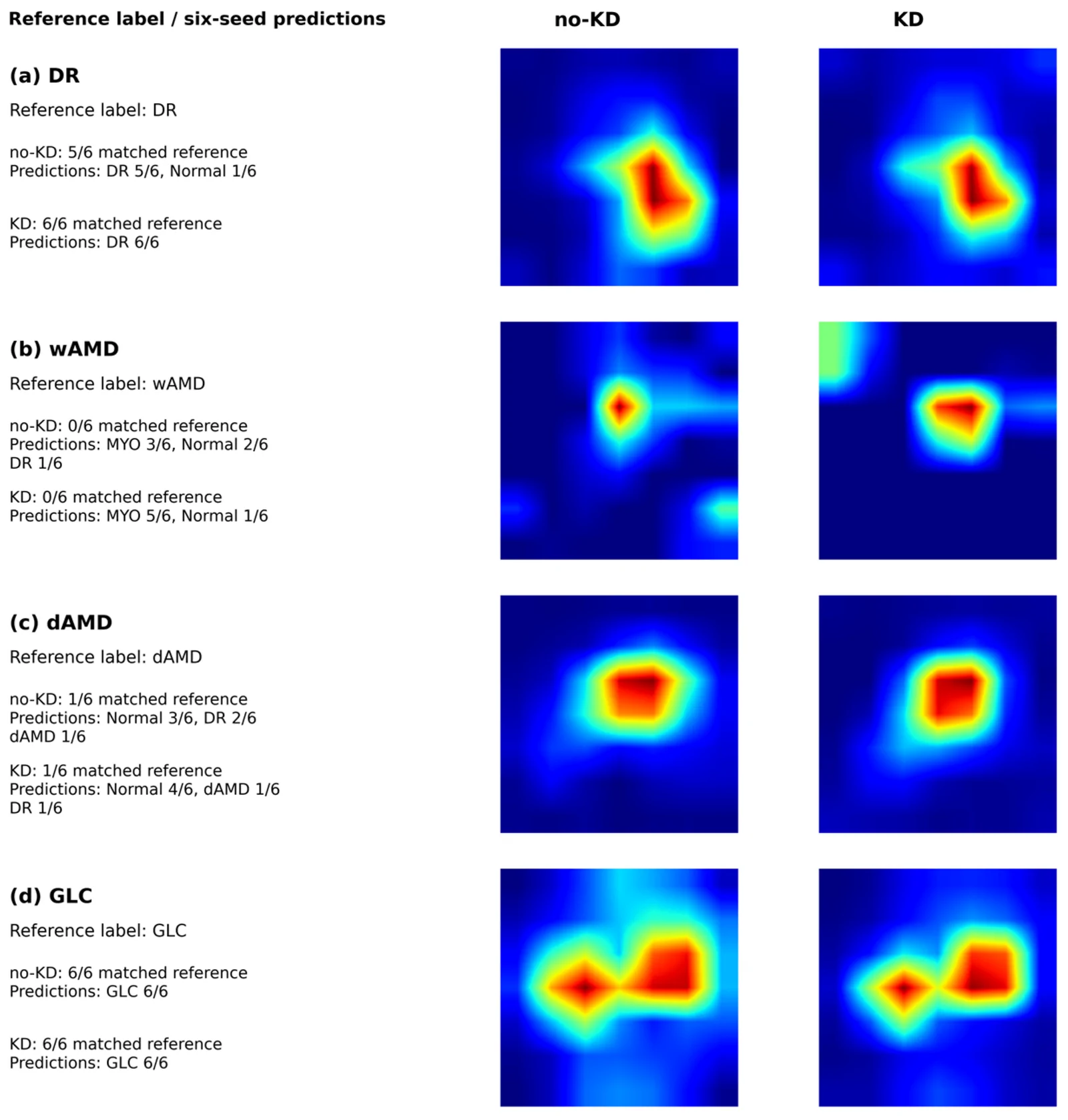
Qualitative Grad-CAM comparison of no-KD and KD EfficientNet-B0 models for four retinal disease classes. One deterministically selected decontaminated TEST case is shown for each of the DR, wAMD, dAMD, and GLC classes. The left column summarizes the reference label and six-seed TEST predictions, while the middle and right columns show the corresponding six-seed consensus Grad-CAM maps for the no-KD and KD models, respectively. Heatmaps were generated with respect to the reference-label class and are intended for qualitative comparison of model-activation patterns.

For presentation in the main manuscript, Figure 2 shows the six-seed consensus Grad-CAM maps for DR, wAMD, dAMD, and GLC, the four non-Normal classes with the largest support in the decontaminated TEST view. For each displayed case, the figure also reports the reference label and the distribution of primary TEST predictions across the six prespecified seeds for the no-KD and KD arms. The complete nine-class no-KD versus KD Grad-CAM comparison is provided in Supplementary Figure S1, while the corresponding direct Grad-CAM++ comparison for the same nine deterministically selected cases is provided in Supplementary Figure S2.

The heatmaps were generated with respect to the raw logit of the reference-label class and therefore represent class-targeted model-activation visualizations rather than explanations necessarily corresponding to the model-predicted class. For each model, the positive native CAM was independently min–max normalized before construction of the six-seed consensus map. Consequently, absolute heatmap intensities are not directly comparable across models, classes, or cases. No lesion-level reference annotations were used for localization validation. Accordingly, the visualizations were interpreted only as qualitative differences in spatial activation patterns and were not considered evidence of improved lesion localization, anatomical superiority of KD over no-KD, or clinical correctness of the highlighted regions.

## 4. Discussion

The present study evaluated whether teacher-guided knowledge distillation could improve a compact multiclass retinal classifier under controlled training and evaluation conditions. KD achieved a higher macro-F1 than the matched no-KD EfficientNet-B0 student, with a mean difference of +0.0394 (95% CI, 0.0252–0.0534; *p* < 0.001). Because the EfficientNet-B0 inference architecture was identical across training conditions, this gain cannot be attributed to increased inference-time model capacity. The findings therefore support KD as a useful training strategy in the present experimental setting, while not establishing clinical superiority or generalizability beyond the evaluated dataset.

The label-smoothing control helps define the scope of this effect. No statistically supported macro-F1 difference was observed between label smoothing and no-KD (Holm-adjusted *p* = 0.554), whereas KD exceeded label smoothing by 0.0360 (Holm-adjusted *p* < 0.001). The evaluated label-smoothing configuration therefore did not reproduce the KD-associated gain. This does not establish the mechanism responsible for the improvement, because the KD arm optimized a composite objective combining weighted supervised learning with temperature-scaled distillation. Class-wise results were also heterogeneous: KD-versus-no-KD F1 point estimates were positive across all nine classes, but only six were statistically supported after correction for multiple comparisons, while the confidence intervals for CSC, MYO, and RVO included zero. The large MEM point estimate should also be interpreted cautiously given its limited TEST support (*n* = 42), while CSC had only 30 TEST records and a wide confidence interval. The overall macro-F1 improvement should therefore not be interpreted as uniform benefit across all retinal conditions.

The probabilistic results were similarly favorable but require careful interpretation. KD had a lower multiclass Brier score than no-KD (difference −0.0767; 95% CI, −0.0812 to −0.0722; Holm-adjusted *p* < 0.001), while ECE decreased descriptively from 0.171 to 0.084. The Brier score reflects overall probabilistic prediction quality rather than calibration alone, and ECE was not subjected to inferential testing. These findings therefore should not be interpreted as statistically established evidence of improved calibration. This distinction is relevant because modern neural networks can produce poorly calibrated confidence estimates [30].

Related retinal studies have used different distillation strategies. The original MultiEYE study used OCT-assisted Conceptual Distillation and reported single-modality fundus accuracies of 78.01% with CLIP and 78.29% with FLAIR [16]. In a supplementary descriptive evaluation on the official MultiEYE TEST partition (*n* = 11,601), the six KD EfficientNet-B0 models in the present study achieved a mean accuracy of 78.32%. These values are provided for context rather than direct performance ranking because the architectures, training procedures, and evaluation protocols differ; moreover, the P-R F1 and S-S F1 measures reported by MultiEYE are not equivalent to the primary macro-F1 endpoint used in the present study. QD-RetNet evaluated cross-modal knowledge distillation on MultiEYE with quantization-aware training [18], whereas the present study used fundus-to-fundus KD without quantization and retained the same EfficientNet-B0 student architecture across training conditions. The 87.7-million-parameter teacher was not required at inference, where only the approximately 4.0-million-parameter student was used; however, this does not establish deployment efficiency because latency, throughput, memory use, and hardware-level performance were not benchmarked in the controlled study.

The explainability analysis was deliberately qualitative. Grad-CAM [19] and direct Grad-CAM++ [20] were applied to one deterministically selected case per class from the decontaminated TEST set, and the principal no-KD versus KD comparison used six-seed consensus maps. Case selection did not depend on model outputs or heatmap appearance. No lesion-level reference annotations were used for localization validation. Accordingly, differences in activation patterns should not be interpreted as evidence that KD localizes retinal lesions more accurately or identifies clinically more appropriate anatomical regions.

Several design features strengthen the within-study comparison, including the matched student architecture, pairing of the training arms within each of six prespecified seeds, prespecified statistical comparisons, and exact-content decontamination. Important limitations remain. The study was conducted on a single benchmark without independent external, prospective, or multicenter validation. Exact-content decontamination does not establish patient-level or eye-level independence or exclude perceptually similar near-duplicates, and the image-level bootstrap does not account for possible unobserved patient- or eye-level clustering. Its uncertainty estimates are therefore conditional on the six trained models. The controlled comparison evaluated one teacher, one KD configuration, and one label-smoothing configuration; alternative imbalance-aware or adaptive-distillation strategies and smaller teachers were not tested. Within the controlled experiment, TEST data were not used for training, hyperparameter tuning, model selection, or protocol modification, and primary TEST evaluation was performed only after all TRAIN/DEV-based controlled decisions had been completed.

Overall, teacher-guided KD improved macro-F1 in a compact multiclass retinal classifier without increasing its inference-time model size. The label-smoothing comparison indicates that the evaluated label-smoothing configuration did not reproduce this gain, while the class-wise findings caution against assuming uniform benefit across retinal diseases. The probabilistic results and qualitative XAI analysis provide complementary information but do not establish clinical superiority, validated lesion localization, or deployment readiness. Independent external validation with patient-level partitioning, where appropriate identifiers are available, and adequate representation of less frequent retinal conditions will be needed to determine whether these findings are reproducible across populations and relevant to clinical use.

## 5. Conclusions

In this controlled nine-class fundus classification study, teacher-guided knowledge distillation improved macro-F1 and lowered the multiclass Brier score compared with an identically structured no-KD EfficientNet-B0 student, while preserving the student’s inference-time architecture and parameter count. Label smoothing did not reproduce the macro-F1 improvement observed with KD, indicating that uniform target smoothing alone did not account for the observed advantage under the evaluated training configuration. However, class-wise effects were heterogeneous, and the findings do not support a uniform benefit across all retinal disease categories.

Overall, the results support knowledge distillation as a potentially useful training strategy for compact multiclass retinal classifiers, but the evidence remains algorithmic and specific to the evaluated setting. Independent patient-level and multicenter validation, particularly in cohorts with greater representation of less frequent retinal diseases, is required to determine reproducibility and generalizability. Prospective clinical evaluation will also be necessary before conclusions regarding diagnostic utility, clinical benefit, or deployment in routine practice can be justified.

## Figures and Tables

**Table 1:** Class distribution of the exact-content-decontaminated MultiEYE fundus views used in the controlled experiments. Values are record counts, with the corresponding share of each partition shown in parentheses.

Class	Training, *n* (%)	DEV, *n* (%)	Test, *n* (%)	Total, *n* (%)
Normal	22,878 (65.76)	7181 (62.23)	6939 (59.96)	36,998 (63.89)
dAMD	1156 (3.32)	84 (0.73)	139 (1.20)	1379 (2.38)
CSC	92 (0.26)	12 (0.10)	30 (0.26)	134 (0.23)
DR	8787 (25.26)	3939 (34.13)	3925 (33.92)	16,651 (28.76)
GLC	625 (1.80)	110 (0.95)	138 (1.19)	873 (1.51)
MEM	160 (0.46)	13 (0.11)	42 (0.36)	215 (0.37)
MYO	327 (0.94)	57 (0.49)	101 (0.87)	485 (0.84)
RVO	367 (1.05)	26 (0.23)	98 (0.85)	491 (0.85)
wAMD	400 (1.15)	118 (1.02)	161 (1.39)	679 (1.17)
Total	34,792 (100.0)	11,540 (100.0)	11,573 (100.0)	57,905 (100.0)

MEM denotes the original MultiEYE class label “Macular Epiretinal Membrane,” corresponding here to epiretinal membrane (ERM). Percentages are column shares within each partition; percentages in the Total column represent the share of the full decontaminated dataset. Percentages may not sum to exactly 100 because of rounding.

**Table 2:** Historical development-stage DEV macro-F1 results from the initial model and hyperparameter screening.

Experiment Group	Model/Setting	DEV Macro-F1	Selection
Student architecture	MobileNetV3-Large + Weighted CE	0.5641	Not selected
Student architecture	EfficientNetV2-B0 + Weighted CE	0.5286	Not selected
Student architecture	EfficientNet-B0 + Weighted CE	0.6208	Selected
Loss function	EfficientNet-B0 + Cross Entropy	0.6059	Not selected
Loss function	EfficientNet-B0 + Weighted Focal Loss	0.5508	Not selected
Teacher architecture	ConvNeXt-Tiny + Weighted CE	0.6690	Not selected
Teacher architecture	ConvNeXt-Small + Weighted CE	0.6880	Not selected
Teacher architecture	RETFound-MAE + Weighted CE	0.6176	Not selected
Teacher architecture	ConvNeXtV2-Base + Weighted CE	0.7098	Selected
KD configuration	EfficientNet-B0 KD, (*T* = 2, *α* = 0.5)	0.6641	Not selected
KD configuration	EfficientNet-B0 KD, (*T* = 4, *α* = 0.3)	0.6566	Not selected
KD configuration	EfficientNet-B0 KD, (*T* = 4, *α* = 0.5)	0.6685	Selected
KD configuration	EfficientNet-B0 KD, (*T* = 4, *α* = 0.7)	0.6578	Not selected

The results in this table were obtained during the historical development stage on the original DEV split before exact-content decontamination and are reported solely to document the model- and hyperparameter-selection process. They are not performance estimates from the subsequent controlled experiment.

**Table 3:** Overall classification performance on the decontaminated TEST view across six seeds. Values are mean ± sample standard deviation.

Metric	no-KD	Label Smoothing	KD
Accuracy	0.7534 ± 0.0036	0.7569 ± 0.0035	0.7830 ± 0.0028
Macro-F1	0.5931 ± 0.0181	0.5965 ± 0.0126	0.6325 ± 0.0135
Weighted-F1	0.7403 ± 0.0043	0.7452 ± 0.0048	0.7715 ± 0.0033
Macro-recall	0.5887 ± 0.0213	0.6040 ± 0.0054	0.6354 ± 0.0144
Macro-precision	0.6490 ± 0.0224	0.6328 ± 0.0219	0.6917 ± 0.0128
Macro-AUROC	0.9124 ± 0.0067	0.8562 ± 0.0081	0.9453 ± 0.0012
Macro-AP	0.6224 ± 0.0218	0.5852 ± 0.0146	0.6688 ± 0.0096

**Table 4:** Class-wise KD-versus-no-KD F1 differences on the decontaminated TEST view.

Class	Support	F1 Difference	95% CI	BH-Adjusted (*p*)
Normal	6939	+0.0214	0.0187 to 0.0239	<0.001
dAMD	139	+0.0434	0.0102 to 0.0770	0.0243
CSC	30	+0.0341	−0.0580 to 0.1215	0.4996
DR	3925	+0.0485	0.0429 to 0.0544	<0.001
GLC	138	+0.0335	0.0043 to 0.0624	0.0388
MEM	42	+0.1365	0.0770 to 0.2015	<0.001
MYO	101	+0.0014	−0.0163 to 0.0200	0.8727
RVO	98	+0.0209	−0.0019 to 0.0439	0.0935
wAMD	161	+0.0154	0.0033 to 0.0290	0.0371

F1 differences are expressed as KD minus no-KD. Confidence intervals were obtained using paired image-level bootstrap resampling. *p*-values were adjusted across all nine class-wise comparisons using the Benjamini–Hochberg false-discovery-rate procedure.

**Table 5:** Prespecified inferential comparisons on the decontaminated TEST view.

Analysis Group	Contrast	Mean Difference	95% CI	*p*-Value	Adjustment
Primary macro-F1	KD − no-KD	+0.0394	0.0252 to 0.0534	<0.001	None
Additional macro-F1 contrasts	Label smoothing − no-KD	+0.0035	−0.0081 to 0.0153	0.554	Holm
Additional macro-F1 contrasts	KD − label smoothing	+0.0360	0.0239 to 0.0479	<0.001	Holm
DR/GLC/Brier comparisons	KD − no-KD, DR F1	+0.0485	0.0429 to 0.0544	<0.001	Holm
DR/GLC/Brier comparisons	KD − no-KD, GLC F1	+0.0335	0.0043 to 0.0624	0.0259	Holm
DR/GLC/Brier comparisons	KD − no-KD, multiclass Brier score	−0.0767	−0.0812 to −0.0722	<0.001	Holm

Mean differences are expressed in the direction indicated by each contrast. Confidence intervals were obtained using paired image-level bootstrap resampling. Holm-adjusted *p*-values were calculated separately within the two additional macro-F1 contrasts and within the three KD-versus-no-KD comparisons comprising DR F1, GLC F1, and multiclass Brier score. For the multiclass Brier score, a negative KD-versus-no-KD difference indicates a lower score with KD.

## Data Availability

The MultiEYE fundus dataset analyzed in this study is available through the official MultiEYE repository on Hugging Face at https://huggingface.co/datasets/Luxuriant16/MultiEYE (accessed on 14 June 2026). No new clinical data were collected or generated by the authors. The results derived from the analyses and supporting the findings of this study are presented within the article. Further information regarding the analyses may be obtained from the corresponding author upon reasonable request.

## Supplementary Materials

The following supporting information can be downloaded at: https://www.mdpi.com/article/10.3390/diagnostics16182945/s1. Figure S1: Nine-class qualitative Grad-CAM comparison of no-KD and KD EfficientNet-B0 models using six-seed consensus maps; Figure S2: Nine-class qualitative direct Grad-CAM++ comparison of no-KD and KD EfficientNet-B0 models using six-seed consensus maps.

## Abbreviations

The following abbreviations are used in this manuscript:

AMD	Age-Related Macular Degeneration
AUROC	Area Under the Receiver Operating Characteristic Curve
CLAHE	Contrast-Limited Adaptive Histogram Equalization
CNN	Convolutional Neural Network
CSC	Central Serous Chorioretinopathy
dAMD	Dry Age-Related Macular Degeneration
DR	Diabetic Retinopathy
ECE	Expected Calibration Error
ERM	Epiretinal Membrane
GLC	Glaucoma
GPU	Graphics Processing Unit
Grad-CAM	Gradient-Weighted Class Activation Mapping
Grad-CAM++	Gradient-Weighted Class Activation Mapping Plus
KD	Knowledge Distillation
KL	Kullback–Leibler
LS	Label Smoothing
MEM	MultiEYE dataset code for epiretinal membrane (ERM)
MYO	Pathological Myopia
RVO	Retinal Vein Occlusion
WCE	Weighted Cross-Entropy
wAMD	Wet Age-Related Macular Degeneration
